# Intersektional und komplex: Aktuelle Forschung und Praxis im Bereich Migration, Flucht und Gesundheit. Welche Aspekte sollten berücksichtigt werden?

**DOI:** 10.1007/s00103-023-03766-5

**Published:** 2023-09-28

**Authors:** Claudia Hövener, Suzan Fiack

**Affiliations:** 1 Abteilung für Epidemiologie und Gesundheitsmonitoring. FG28 Soziale Determinanten der Gesundheit, Robert Koch-Institut, Nordufer 20, 13302 Berlin, Deutschland; 2 Bundesinstitut für Risikobewertung, Max-Dohrn-Str. 8–10, 10589 Berlin, Deutschland

Deutschland ist ein Einwanderungsland, was sich in verschiedenen Migrationsbewegungen zeigt. Beispiele sind die Arbeitsmigration seit den 1950er-Jahren und im Rahmen der EU-Freizügigkeitsabkommen sowie die Fluchtmigration aufgrund von Kriegen und politischen Konflikten. Menschen mit Einwanderungsgeschichte, also Personen, die selbst oder deren beide Eltern nach Deutschland migriert sind, machten im Jahr 2022 insgesamt 24,3 % der in Deutschland lebenden Bevölkerung aus [1]. Von diesen ca. 20,2 Mio. Menschen waren 15,3 Mio. selbst eingewandert.

Die Gruppe der Menschen mit Einwanderungsgeschichte ist äußerst divers, z. B. in Bezug auf Gründe der Migration, Aufenthaltsdauer und -status, Lebensumstände und Teilhabechancen. Diese Heterogenität gilt es bei der Beschreibung der gesundheitlichen Lage mit zu berücksichtigen [2]. Eine eigene oder familiäre Migrationserfahrung an sich macht demnach nicht krank oder gesund. Allerdings gibt es verschiedene Aspekte vor, während und nach dem Migrationsprozess, die den Gesundheitszustand beeinflussen können [3].

In Bezug auf die Zeit vor und während des Migrationsprozesses macht es einen Unterschied, ob Personen aufgrund von Krieg oder politischen Konflikten fliehen und lange, unsichere, prekäre Migrationswege nach Deutschland auf sich nehmen (müssen) oder ob sie mit einer gesicherten Bleibeperspektive über einen sicheren Weg nach Deutschland migrieren. Ersteres ist laut Studien mit höheren Prävalenzen von z. B. posttraumatischen Belastungsstörungen und Depressionen assoziiert [4, 5]. In Deutschland, also nach dem Migrationsprozess, unterscheiden sich Menschen mit (familiärer) Migrationserfahrung in Bezug auf verschiedene Faktoren, wie ihren Aufenthaltsstatus, ihre sozioökonomische Situation, Kenntnisse der deutschen Sprache sowie Teilhabechancen. All diese Faktoren sind mit unterschiedlichen gesundheitlichen Chancen, Risiken und Versorgungsbedarfen assoziiert und sollten im Rahmen der Berichterstattung zu Migration, Flucht und Gesundheit berücksichtigt werden. Nur so können differenzierte Aussagen zur gesundheitlichen Lage von Menschen mit Migrationserfahrung gemacht werden, die spezifische Benachteiligungen, Vulnerabilitäten und somit auch Ansatzpunkte für Interventionen identifizieren.

Das bedeutet, dass wir in der Forschung und Praxis Migration und Flucht als *eine* relevante Determinante von Gesundheit betrachten, die aber mit vielen anderen sozialen und ökonomischen Bedingungen interagiert, wie z. B. dem Einkommen, dem Zugehörigkeitsgefühl zur Gesellschaft und Diskriminierungserfahrungen [2, 6]. Praktisch heißt das, dass wir uns von groben Kategorien wie „Migrationserfahrung“ vs. „keine Migrationserfahrung“ verabschieden, um im Sinne eines intersektionalen Verständnisses die Lebenswelten von Menschen mit (familiärer) Migrationserfahrung zu berücksichtigen diese als Ansatzpunkt für die praktische Arbeit zu nutzen und so gesundheitliche Ungleichheiten gezielt identifizieren und angehen zu können.

Das vorliegende Themenheft „Migration, Flucht und Gesundheit – Aktuelle Perspektiven aus Deutschland“ hat zum Ziel, aktuelle Erkenntnisse zu Forschung, Konzepten und Praxiserfahrungen zu bündeln und unterteilt sich in 3 Bereiche: 1) Forschungsstand, 2) theoretische Aspekte zum Zusammenhang von Migration und der Intersektion mit anderen sozialen Determinanten in Bezug auf Gesundheit und 3) Gesundheitsversorgung und Praxis der Gesundheitsförderung mit Menschen mit Migrationserfahrung.

## 1) Forschungsstand.

Zunächst wird im Rahmen von zwei Beiträgen ein Überblick zum nationalen Forschungsstand zur Gesundheit von Menschen mit Migrationsgeschichte und Geflüchteten gegeben. Der Beitrag von *Bartig und Bug et al.* fokussiert auf die Beschreibung der gesundheitlichen Lage in Bezug auf nichtübertragbare Erkrankungen von Menschen mit ausgewählten Staatsangehörigkeiten sowie die Identifikation relevanter Einflussfaktoren. Der Artikel von *Führer *stellt im ersten Teil strukturelle Determinanten dar, die den Zugang Asylsuchender zur Versorgung erschweren bzw. beeinflussen. Im zweiten Teil wird die gesundheitliche Lage Asylsuchender mit der gesetzlich Versicherter verglichen.

## 2) Theoretische Aspekte zum Zusammenhang von Migration und der Intersektion mit anderen sozialen Determinanten in Bezug auf Gesundheit.

Im Rahmen von konzeptionellen Erklärungsmodellen zu Migration und Gesundheit zeigt der Beitrag von *Spallek et al.* die Potenziale der Lebenslaufepidemiologie für die Migrationsforschung auf. Der Artikel von *Kajikhina et al.* fokussiert auf die strukturellen Aspekte von Rassismus sowie deren direkte und indirekte Intersektionen mit gesundheitlicher Ungleichheit. Abschließend geht der Beitrag von *Akbulut und Razum* auf „Othering“ am Beispiel Migration ein und beschreibt anhand praxisbezogener Ergebnisse die Auswirkungen des Othering auf die Gesundheitsversorgung. Unter Othering verstehen die Autor*innen „einen gesellschaftlichen Prozess, der Unterschiede so konstruiert und klassifiziert, dass bestimmte Gruppen als wesentlich Andere sozial sichtbar werden“. 

## 3) Gesundheitsversorgung und Praxis der Gesundheitsförderung.

Das Heft schließt mit Beiträgen aus der praktischen Arbeit in den Bereichen Gesundheitsversorgung und -förderung und beginnt mit einem Beitrag von *Nowak und Hornberg *zu Erfahrungen von Menschen mit Fluchtgeschichte in Bezug auf Gesundheitsversorgung und der Ableitung von Handlungsstrategien, um Herausforderungen zu adressieren. Der Artikel von *Dimitrova und Sehouli* stellt die Bedeutung interkultureller Kompetenz für das Gesundheitswesen dar und beleuchtet, welche dieser Aspekte für die medizinische Versorgung besonders relevant sind. Abschließend folgt ein Beitrag von *Führer et al.*, der das „Lehrnetzwerk Migration und Gesundheit“ vorstellt, welches „eine menschenrechtsbasierte, diversitätssensible und Equity-orientierte Weiterentwicklung“ von Curricula zum Ziel hat.

Die Beiträge zeigen eindrucksvoll auf, dass gesundheitliche Ungleichheiten im Kontext von Migration und Flucht komplex sind und dass eine differenzierte Betrachtungsweise und die Berücksichtigung verschiedener anderer sozialer Determinanten nötig sind, um konkrete Ansatzpunkte für die bedarfsorientierte Anpassung von Angeboten zur Gesundheitsversorgung, Prävention und Gesundheitsförderung abzuleiten. Hier ist es essentiell, individuelle und strukturelle Barrieren in den Blick zu nehmen und lebensweltorientiert vorzugehen. Die Erkenntnisse zeigen einmal mehr, dass Migrationserfahrung an sich nicht kränker oder gesünder macht, sondern, dass wir die Lebensbedingungen, Diskriminierungserfahrungen und Teilhabechancen der Menschen in den Vordergrund stellen müssen. Wir sehen aber auch, dass noch viel Arbeit vor uns liegt, um intersektional gesundheitliche Ungleichheiten im Bereich Migration und Flucht aufzeigen zu können. Wir danken Frau Dr. Anke Spura für ihre wertvolle Mitarbeit bei der Konzeption dieses Themenhefts.
